# Editorial: APOBEC and ADAR in oncogenesis: from molecular mechanisms to therapeutic targets

**DOI:** 10.3389/fgene.2026.1827626

**Published:** 2026-04-10

**Authors:** Xiaoling Li, Yunguan Wang, Ping Mu

**Affiliations:** 1 Department of Urology, Yale School of Medicine, New Haven, CT, United States; 2 Yale Cancer Center, Yale University, New Haven, CT, United States; 3 Division of Pediatric Gastroenterology, Hepatology and Nutrition, Cincinnati Children's Hospital Medical Center, Cincinnati, OH, United States; 4 Department of Pediatrics, University of Cincinnati, Cincinnati, OH, United States; 5 Department of Pathology, Yale School of Medicine, New Haven, CT, United States

**Keywords:** ADAR, APOBEC, cancer therapeutics, molecular targeting in cancer, oncogenic mechanisms

In the changing world of cancer genomics, the relationship between APOBEC (Apolipoprotein B mRNA Editing Enzyme, Catalytic Polypeptide-Like) and ADAR (Adenosine Deaminases Acting on RNA) enzymes has emerged as critical factors. These enzymes are primarily involved in DNA mutation and RNA editing processes, contributing significantly to genetic variation and cellular instability. Their dysregulation is a known driver in the development and progression of various cancer types, offering both a window into cancer biology and potential new strategy for targeted therapy. This Research Topic has four contributions that look at the molecular mechanisms of these enzymes and evaluates their clinical significance across different cancer types.

The two contributions most central to APOBEC and ADAR are those by Lehle et al. and Granadillo Rodriguez et al., which provide critical mechanistic and functional perspectives on the APOBEC3 (A3) family. Lehle et al. provide a comprehensive review of the interplay between viral infection and A3 dysregulation. A3 enzymes serve as a critical frontline defense in the host’s innate immune system, where they typically protect cellular integrity by inducing mutations in viral DNA. However, infection by oncogenic viruses, including HPV, HBV, EBV, and HPyV can chronically dysregulate these enzymes through inflammatory and interferon-signaling pathways. This antiviral response inadvertently targets host single-stranded DNA during replication, generating extensive mutations with landscapes correspondent to the COSMIC SBS2/13 mutational signatures in over 50% of tumor genomes. Such ectopic genomic instability could ultimately fuel the progression of various human malignancies. The authors emphasize the “hit-and-run” hypothesis, suggesting that viruses may initiate genomic damage through A3 enzymes and then disappear, a phenomenon that complicates the diagnosis and treatment of virus-associated cancers, complicating efforts to establish clear links between infection and A3-induced mutagenesis. This work underscores the dual role of A3 enzymes as both viral defenders and potential genomic threats.

Providing essential experimental clarity, Granadillo Rodriguez et al. use a breast cancer mouse xenograft model to differentiate the functional impacts of specific A3 isoforms. While A3B has long been considered the primary source of APOBEC mutational signatures in breast cancer due to its high expression, this study demonstrates that A3A and A3H Haplotype I are more likely to enhance tumor progression, whereas high level of A3B actually decreased tumor size in this model by downregulating protein synthesis and cell cycle pathways. This study underscores that the functional consequences of A3 activity are unique to each enzyme and do not always correlate directly with the number of mutations they induce. These results emphasize that the impact of A3 enzymes is unique and that simply counting mutations is insufficient to understand their full functional role in cancer evolution.

Complementing these APOBEC-focused studies, Zhang et al. broaden the context to include ADAR-mediated RNA editing in bladder cancer. By integrating 34 “writer” enzymes across five modification types, including A-to-I editing by ADAR, they developed an RNA modification “writers” score (RMS). Their study identifies two distinct RNA modification patterns with different clinical outcomes. The RMS model proved to be a robust prognostic indicator, with high scores correlating with immunosuppressive cell infiltration and the activation of oncogenic pathways like EMT and angiogenesis. Crucially, the RMS model differentiated responders from non-responders in immunotherapy, highlighting the potential of RNA modification signatures to guide clinical intervention.

Finally, Xu et al. investigate the clinical impact of ubiquitination, a key post-translational modification, by developing a six-gene prognostic transcriptomic signature (ARHGAP4, MID2, SIAH2, TRIM45, UBE2D2, and WDR72) for colon cancer. This model effectively stratifies patients into risk groups, where high-risk cases are characterized by enhanced epithelial-mesenchymal transition (EMT) and an immunosuppressive microenvironment featuring high levels of myeloid-derived suppressor cells (MDSCs) and regulatory T cells. Conversely, the low-risk group demonstrates higher immunogenicity and a better response to CTLA4 checkpoint inhibitors. Through functional validation, the authors also highlight WDR72 as a driver of tumor cell proliferation, underscoring how post-translational regulation through the ubiquitin system shapes the colorectal cancer landscape and dictates therapeutic sensitivity.

Collectively, the articles in this Research Topic illustrate the complex roles of APOBEC and ADAR in oncogenesis. From the development of prognostic models like the RMS to the identification of viral triggers and the functional mapping of specific A3 isoforms, these studies broaden our context of how these enzymes contribute to the cancer landscape. As our understanding of these molecular mechanisms deepens, the transition from laboratory findings to effective, personalized cancer therapies becomes an increasingly attainable goal.

